# Purification of human respiratory syncytial virus by ultracentrifugation in iodixanol density gradient

**DOI:** 10.1016/j.jviromet.2007.09.013

**Published:** 2008-02

**Authors:** E. Gias, S.U. Nielsen, L.A.F. Morgan, G.L. Toms

**Affiliations:** The School of Clinical Medical Sciences, The Medical School, The University of Newcastle upon Tyne, Newcastle upon Tyne NE2 4HH, United Kingdom

**Keywords:** Human respiratory syncytial virus, PEG concentration, Iodixanol, Purification, Ultracentrifugation

## Abstract

Ultracentrifugation in sucrose density gradient remains the most commonly used technique for hRSV purification. However, the high viscosity and hyper-osmotic property of sucrose can cause damage to the extremely labile virus leading to loss of infectivity. To overcome these limitations, an alternative purification technique was developed using iodixanol as gradient medium, incorporating MgSO_4_ as a stabilizing agent and EDTA to disaggregate the virus prior to infectivity assay. Virus particles were banded at the 20–36% interface after purification of polyethylene glycol-concentrated viruses by rate zonal ultracentrifugation on a 20–52% discontinuous iodixanol gradient. The presence of the virus was confirmed by viral fusion glycoprotein content using ELISA. After further purification by buoyant density ultracentrifugation on a 20–52% continuous gradient, the virus was recovered in the region of density 1.15–1.19 g/ml and this was confirmed by the coincidence of the infectivity titre, viral genome and fusion glycoprotein peaks. Analysis of recovery rates showed that the use of iodixanol increased the virus yield up to 69%. Iodixanol was also found to be non-toxic to HeLa cells used in infectivity assay, eliminating the need of its downstream removal by dialysis.

## 1 Introduction

Human respiratory syncytial virus (hRSV) is an enveloped, single stranded, negative sense RNA virus belonging to the genus *Pneumovirus* and family *Paramyxoviridae* (Fauquet et al., 2005). It is a medically important paediatric virus responsible for acute respiratory illnesses worldwide (Weber et al., 1998). The virus particle is composed of a helical nucleocapsid enclosed by a lipid envelope studded with three transmembrane glycoproteins, G, F and SH (Collins et al., 2001). While the function of SH protein is yet unknown, the G and F glycoproteins mediate virus attachment and fusion, respectively, and are major targets of the protective host immune response (Elango et al., 1986; Routledge et al., 1988; Walsh et al., 1998).

Purified hRSV preparations are often required for characterization and immunological studies. Multiple steps of differential and density gradient ultracentrifugation have commonly been used to purify hRSV, a procedure which takes many hours and can result in considerable loss of infectivity for this fragile virus. The recovery rates have been improved by the addition of MgSO_4_ to the buffer during purification (Fernie and Gerin, 1980) and Ueba (1978) reported that sucrose in concentrations above 15% also has a stabilizing effect. Mbiguino and Menezes (1991) found that fractionation on sucrose gradients gave better virus recovery than percoll, renografin and metrizamide gradients. However, ultracentrifugation in sucrose is damaging to virus particles (Trepanier et al., 1981) and yields remain variable and often unsatisfactory. For example, although Mbiguino and Menezes (1991) report recovery of 57%, Ueba (1978) recovered 19% and Trepanier et al. (1981) 22% of virus after centrifugation through two successive sucrose density gradients. In addition to poor recovery of infectivity, sucrose is also toxic to cells used to grow the virus and this affects downstream experimental work which requires the use of cell cultures such as viral infectivity assays.

Iodixanol (OptiPrep™) has been used successfully in the purification of various viruses such as HIV-1, HTLV-1, recombinant adeno-associated virus (rAAV), MoMLV-derived retrovirus particles (Dettenhofer and Yu, 1999; Hermens et al., 1999; Moller-Larsen and Christensen, 1998; Segura et al., 2006) and hepatitis C virus (Nielsen et al., 2006). Iodixanol offers several advantages over sucrose. The medium is less viscous than sucrose and non-toxic to cells, allowing subsequent viral infectivity assays to be carried out directly without the need for removal. Iodixanol also has a low osmolarity and therefore can be diluted in iso-osmotic buffers to form iso-osmotic solutions. This allows the virus to be purified under iso-osmotic conditions and thus better preserve the integrity and functionality of the virus particles (Axis-Shield, 2004).

In the present study, the use of iodixanol was investigated as a medium for purifying hRSV in an attempt to obtain an improved recovery rate of intact and infectious virion particles. A purification procedure was developed based on the method of ultracentrifugation in sucrose gradients described by Mbiguino and Menezes (1991).

## 2 Materials and methods

### 2.1 Virus

hRSV strains R17532 (1994) and N5843 (1990) were isolated from infected infants in Newcastle upon Tyne and passaged three and one times, respectively, in HeLa cells. Stocks were prepared as described in Section 2.2 after two rounds of biological cloning in Vero cells. The hRSV strain A2 was kindly supplied by Dr A.J. Stott, Institute of Animal Health, Compton, UK.

### 2.2 Cell culture

Human cervical carcinoma (HeLa) cells were cultured in a 225 cm^2^ cell culture flask (Corning Incorporated, USA) in Eagle's minimal essential medium supplemented with 10% foetal calf serum (FCS), 10 mg/ml of penicillin-streptomycin, 1% of 200 mM L-glutamine and 5% gassed sodium bicarbonate containing 0.4% phenol red. The virus-infected cells were maintained in Medium 199 Earles Salts containing 2% of FCS.

Confluent HeLa cell cultures infected with each virus strain were harvested when cytopathic effect involved the whole monolayer, snap-frozen in liquid nitrogen and stored at −80 °C. On thawing, the infected cell suspensions were clarified by centrifugation in a JLA 10.5 rotor (Beckman Coulter, USA) at 3250 × *g* for 20 min at 4 °C. The clarified viral supernatants were then adjusted to 100 mM MgSO_4_ and 50 mM Tris–HCl (pH 7.5).

### 2.3 Virus concentration

Fifty percent (w/v) PEG 6000 in NT buffer (150 mM NaCl, 50 mM Tris–HCl, pH 7.5) was added to the clarified culture fluid to a final concentration of 10% (v/v). The virus particles were then precipitated for 90 min at 4 °C with moderate stirring, followed by centrifugation at 3250 × *g* for 20 min at 4 °C in a JLA 10.5 rotor. After removing the supernatant, the pellet was re-centrifuged as above and the residual fluids were removed. The pellet was then re-suspended in 1 ml of cold NT buffer. A sample was taken out and EDTA was incorporated to a final concentration of 1 mM and the infectivity titre was determined by the infectious focus assay in HeLa cell monolayers as described by Routledge et al. (1988). Eighteen hours after infection the infected monolayers were fixed with 75% acetone in phosphate buffered saline. Infectious foci were visualised by indirect immunofluorescence with a pool of monoclonal antibodies to hRSV (Novocastra Laboratories Ltd., Newcastle upon Tyne, UK) followed by fluorescein conjugated goat anti-mouse IgG (Dako UK Ltd., Ely, UK) and counted under a fluorescence microscope.

### 2.4 Purification by ultracentrifugation

Discontinuous gradients were prepared by sequentially layering 52, 36 and 20% iodixanol solution (OptiPrep™, Axis-Shield PoC AS, Oslo, Norway) in 10 mM Tris–HCl, pH 7.5, 100 mM MgSO_4_ and 0.25 M sucrose into 14 ml centrifuge tubes (Beckman Coulter UK Ltd., High Whickham, Bucks, UK), and the gradients were used immediately. Continuous gradients containing iodixanol solution to final concentrations of 52, 42, 32 and 20% were prepared in the same manner but left in the dark at 4 °C overnight to allow the gradient to diffuse. PEG-concentrated virus samples were adjusted to 100 mM MgSO_4_, layered over a discontinuous gradient and centrifuged in a SW-40 rotor (Beckman Coulter) for 90 min at 35,000 rpm at 4 °C. An opaque virus band was visible at the 20–30% interface. Fractions were harvested and the opaque band was recovered and diluted 1:2 in cold NT buffer containing 100 mM MgSO_4_. The diluted virus band was layered over the continuous gradient and centrifuged in a SW-40 rotor for 18 h at 35,000 rpm at 4 °C. After 18 h, gradient fractions were harvested and the densities of fraction samples were measured using a refractometer (ATAGO, Tokyo, Japan) after which they were snap-frozen in liquid nitrogen and stored at −80 °C. The presence of hRSV in fraction samples was determined by MAb-capture ELISA and quantitative RT-PCR (see below), and the infectivity titres were determined by the infectious focus assay as described above in the presence of 1 mM EDTA.

### 2.5 Monoclonal antibody (MAb)-capture ELISA

The hRSV F glycoprotein in gradient fractions was detected by MAb-capture ELISA as described by Morgan (1988). Virus samples were serially diluted 2-fold in PBS containing 0.05% Tween 20 and 10% foetal calf serum. 50 μl per well of samples and control antigens (non-infected and hRSV strain A2 infected HeLa cells) followed by 20 μl of antigen treatment buffer (20 mM of NaC1, 0.72 mM of KH_2_PO_4_, 4 mM Na_2_HPO_4_·12H_2_O and 1.3 mM of KCl) were added to the 96-well Maxisorp Nunc-Immunoplate plates (Nunc™, Roskilde, Denmark) coated with 1 in 400 dilution of anti-F MAb IE3 ascites and incubated at 37 °C for 2 h. The presence of hRSV antigen was detected by a 1 in 600 dilution of a polyclonal rabbit anti-hRSV serum for 1 h followed by 1 in 1000 dilution of peroxidase-conjugated swine immunoglobulins to rabbit IgG (Dako) for another hour. 100 μl per well of freshly prepared complete substrate solution containing 62.5 mg/ml of OPD, 0.02 M of citric acid, 0.06 M of Na_2_HPO_4_ and 0.01% H_2_O_2_ was added, incubated at 37 °C for 30 min and the colour development was stopped by the addition of 100 μl per well of 3M H_2_SO_4_. The optical density of each well was then measured using an MRX plate reader at an absorbance of 492 nm. To test the specificity of the assay, the rabbit polyclonal anti-hRSV antibody/anti-rabbit peroxidise detector system was replaced with monoclonal antibodies to the hRSV F, G and N proteins and peroxidase labelled anti-mouse immunoglobulins (Abcam Plc, Cambridge, UK). While anti-F MAb bound well in this system, the binding of MAbs to other viral proteins was negligible (data not shown).

### 2.6 Quantitative reverse transcriptase polymerase chain reaction (RT-PCR) for hRSV genome

The amount of hRSV genome in fraction samples was quantified by real-time PCR based on the protocol described by Dewhurst-Maridor et al. (2004). Samples to be quantified were diluted 1/64 in diethyl pyrocarbonate (DEPC) treated distilled water containing carrier RNA. Reverse transcription was carried out in a 24-well plate (Applied Biosystems, Warrington, UK) in 30 μl volumes containing 10 μl of RNA, 5 mM MgCl_2_, 1× reverse transcription buffer, 1 mM dNTPs, 2.75 μM of RSV AF primer (Dewhurst-Maridor et al., 2004), 30 units RNasin Ribonuclease inhibitor, 5 units AMV-RT and DEPC-treated distilled water. Synthesis of cDNA was carried out in a PTC-200 thermocycler (MJ Research Inc., USA) at 42 °C for 30 min and 99 °C for 5 min. Real-time PCR was carried out in a 48-well optical PCR plate (Applied Biosystems, Warrington, UK) in 30 μl volumes containing 6 μl of cDNA, 1× TaqMan Universal PCR Master Mix, 200 nM of RSV probe A, 600 nM of each RSV AF and RSV AR primers (Dewhurst-Maridor et al., 2004) and DEPC-treated distilled water. Real-time PCR was carried out in ABI Prism 7000 (Applied Biosystems, Warrington, UK) using the following conditions: initial denaturation at 95 °C for 10 min, and 45 cycles of 95 °C for 15 s, 55 °C for 45 s and 60 °C for 1 s. The result of amplification was analyzed using ABI-PRISM 7000 SDS software. A standard curve was generated from three fold dilutions of RNA-extracted from a HeLa cell culture infected with hRSV strain A2 arbitrarily attributed 10^6^ genome/100 μl. The hRSV RNA content in test samples is expressed in arbitrary units derived by comparison with the standard curve.

## 3 Results

**Fig. 1:**
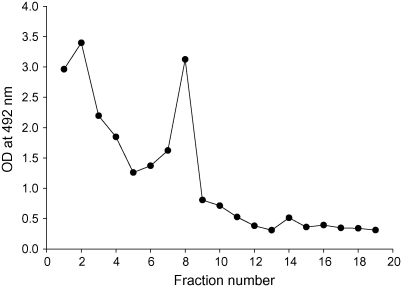
Purification of PEG-concentrated hRSV strain R17532 on discontinuous iodixanol gradient. The amount of viral F glycoprotein in gradient fractions was determined by ELISA. The peak fraction 8 was diluted 1:2 in NT buffer containing MgSO_4_ and layered on a continuous iodixanol gradient.

hRSV R17532 and N5843 viruses from clarified, infected cell lysates were concentrated by PEG precipitation and purified from the precipitates by sequential ultracentrifugation on discontinuous and continuous iodixanol gradients. After centrifugation of PEG-concentrated virus through discontinuous iodixanol gradients, a visible opaque band was observed for both virus strains at the 20–36% interface. In order to confirm the presence of virus particles in these bands, samples from all fractions of the discontinuous gradient of strain R17532 were measured for the amount of viral F glycoprotein by ELISA. A peak of viral glycoprotein content was found to coincide with fraction 8 which contained the opaque band (Fig. 1). A second peak of viral antigen was also detected in fraction 2 at a density of 1.10 g/ml and may represent microsomal membrane containing viral glycoproteins or cell-associated infectious virus.

**Fig. 2:**
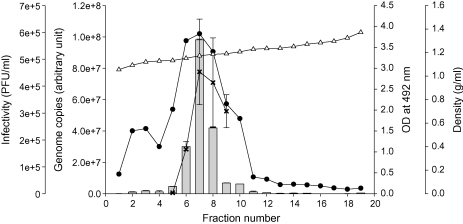
Purification of hRSV strain R17532 on continuous iodixanol gradient. The density (g/ml, Δ), amount of hRSV genome by real-time PCR () and F glycoprotein by ELISA (●) was determined across the fractions. The infectivity titres (PFU/ml, x) were determined for fractions 5–9 only.

Centrifugation of the fraction 8 band of virus on continuous gradients yielded a single opaque band for both virus strains at similar positions. In order to examine the distribution of virus in the gradients, viral infectivity, the amount of viral glycoprotein and genomic RNA were analysed by infectious focus assay, MAb capture ELISA and quantitative RT-PCR, respectively, in all fractions samples of virus strain R17532. The distribution of viral genome was found to be consistent with that of the F glycoprotein across the gradient, and the increase of viral genome was proportional with the infectivity titres in fractions 5–9 (Fig. 2). The coincidence of the F glycoprotein, viral genome and infectivity peaks confirmed the presence of virus particles, in the region of density 1.15–1.19 g/ml. Similar results were also obtained with strain N5843 and the density of the peak fraction of the two virus strains averaged 1.16 ± 0.02 g/ml. The recovery rate of the virus from fractions containing peak amounts of F glycoprotein and genomic RNA ranged from 66 to 71%, with an average of 69% (Table 1).

## 4 Discussion

As hRSV generally has a low infectivity titre after propagation in cell culture, for purification, large volumes of cell culture fluids are required in order to achieve high virus titres in the purified product. Here, virus in clarified culture supernatants was concentrated by PEG precipitation and resuspended in NT buffer containing 100 mM MgSO_4_. MgSO_4_ was incorporated into the procedure to stabilize the virus (Fernie and Gerin, 1980). A preliminary comparative study confirmed that the infectivity titre of virus purified in the presence of MgSO_4_ was 8-fold higher than that without MgSO_4_ (data not shown).

Ultracentrifugation of PEG concentrates through discontinuous iodixanol density gradients produced two peaks of material detected by optical density. Viral F protein peaked at the 20–36% interface, with a calculated density of 1.10–1.20 g/cm^3^, and this corresponds to the previous 30–45% sucrose interface containing virus band (Mbiguino and Menezes, 1991; Ueba, 1978). The second peak at the 45–60% interface (1.20–1.29 g/ml) is thought to contain host material. A similar optical density peak was present in the discontinuous gradients of Mbiguino and Menezes who, as here, fractionated virus derived from infected cell lysates, but was absent from those of Ueba who used only clarified culture superntants to minimise the host cell debri in the starting material.

On the subsequent continuous gradient, both strains of hRSV were banded at 1.15–1.19 g/ml, a different density to that of hRSV banded on sucrose at 1.18–1.20 g/ml. This finding is consistent with that of Mbiguino and Menezes (1991) who reported variable virus densities with different purification media. The yield of both virus strains was approximately 70% after the complete purification procedure, which is somewhat higher than the rate reported previously for sucrose gradients (Mbiguino and Menezes, 1991; Ueba, 1978). Consistent with the findings of these authors, the infectivity titre was improved by incorporating EDTA (1 mM) to the fraction samples prior to the infectivity assay to disaggregate the virus (data not shown). Unlike sucrose, iodixanol is not toxic to HeLa cells and preliminary experiments have shown that it has no effect on the viral titre (data not shown), allowing the infectivity assays to be carried out directly on the purified samples without dialysis.

In conclusion, the results of the present study show that iodixanol density gradient centrifugation yields high recovery of purified infectious hRSV particles. The method is particularly suited to the purification of early passage isolates which produce low virus titers after propagation in cell culture.

## Figures and Tables

**Table 1:** Recovery rates of hRSV from continuous iodixanol gradients

	PEG-precipitated virus			Continuous gradient				
	Volume^a^ (ml)	PFU/ml^a^ (×10^4^)	Total PFU^a^ (×10^4^)	Density (g ml^−1^)	Volume (ml)	PFU/ml^a^ (×10^4^)	Total PFU^a^ (×10^4^)	Recovery (%)
R17532	0.8	64 ± 5.66	51.2 ± 4.52	1.15–1.19	1.5	24.35 ± 17.66	36.52 ± 35.92	71
N5843	0.7	3.23 ± 0.24	2.26 ± 0.16	1.15–1.16	0.9	1.66 ± 0.93	1.49 ± 0.30	66

a Mean of two gradients, ±Standard deviation.

## References

[bib1] Axis-Shield, P.A., 2004. Density Gradient Media: Application and Products 2005, Oslo, Norway, http://www.axis-shield.com/densityhome/density/dhome.htm.

[bib2] Collins P., Chanock R., Murphy B., Knipe D., Howley P. (2001). Respiratory syncytial virus.

[bib3] Dettenhofer M., Yu X.F. (1999). Highly purified human immunodeficiency virus type 1 reveals a virtual absence of Vif in virions. J. Virol..

[bib4] Dewhurst-Maridor G., Simonet V., Bornand J.E., Nicod L.P., Pache J.C. (2004). Development of a quantitative TaqMan RT-PCR for respiratory syncytial virus. J. Virol. Methods.

[bib5] Elango N., Prince G.A., Murphy B.R., Venkatesan S., Chanock R.M., Moss B. (1986). Resistance to human respiratory syncytial virus (RSV) infection induced by immunization of cotton rats with a recombinant vaccinia virus expressing the RSV G glycoprotein. Proc. Natl. Acad. Sci. USA.

[bib6] Fauquet C., Mayo M., Maniloff J., Desselberger U., Ball L. (2005). Virus Taxonomy: VIIIth Report of the International Committee on Taxonomy of Viruses.

[bib7] Fernie B.F., Gerin J.L. (1980). The stabilization and purification of respiratory syncytial virus using MgSO_4_. Virology.

[bib8] Hermens W.T., ter Brake O., Dijkhuizen P.A., Sonnemans M.A., Grimm D., Kleinschmidt J.A., Verhaagen J. (1999). Purification of recombinant adeno-associated virus by iodixanol gradient ultracentrifugation allows rapid and reproducible preparation of vector stocks for gene transfer in the nervous system. Hum. Gene Ther..

[bib9] Mbiguino A., Menezes J. (1991). Purification of human respiratory syncytial virus: superiority of sucrose gradient over percoll, renografin, and metrizamide gradients. J. Virol. Methods.

[bib10] Moller-Larsen A., Christensen T. (1998). Isolation of a retrovirus from multiple sclerosis patients in self-generated Iodixanol gradients. J. Virol. Methods.

[bib11] Morgan, L., 1988. Respiratory syncytial virus antigen immunoassay. PhD thesis, Department of Virology, The University of Newcastle upon Tyne, Newcastle upon Tyne.

[bib12] Nielsen S.U., Bassendine M.F., Burt A.D., Martin C., Pumeechockchai W., Toms G.L. (2006). Association between hepatitis C virus and very-low-density lipoprotein (VLDL)/LDL analyzed in iodixanol density gradients. J. Virol..

[bib13] Routledge E.G., Willcocks M.M., Samson A.C., Morgan L., Scott R., Anderson J.J., Toms G.L. (1988). The purification of four respiratory syncytial virus proteins and their evaluation as protective agents against experimental infection in BALB/c mice. J. Gen. Virol..

[bib14] Segura M.M., Garnier A., Kamen A. (2006). Purification and characterization of retrovirus vector particles by rate zonal ultracentrifugation. J. Virol. Methods.

[bib15] Trepanier P., Payment P., Trudel M. (1981). Concentration of human respiratory syncytial virus using ammonium sulfate, polyethylene glycol or hollow fiber ultrafiltration. J. Virol. Methods.

[bib16] Ueba O. (1978). Respiratory syncytial virus. I. Concentration and purification of the infectious virus. Acta Med. Okayama.

[bib17] Walsh E.E., Falsey A.R., Sullender W.M. (1998). Monoclonal antibody neutralization escape mutants of respiratory syncytial virus with unique alterations in the attachment (G) protein. J. Gen. Virol..

[bib18] Weber M.W., Mulholland E.K., Greenwood B.M. (1998). Respiratory syncytial virus infection in tropical and developing countries. Trop. Med. Int. Health.

